# Research on the Elastic–Plastic Behaviors of Bicontinuous Polymer Matrix and Carbon Fiber-Reinforced Composites Based on Micromechanical Modelling

**DOI:** 10.3390/polym17182517

**Published:** 2025-09-17

**Authors:** Bin Yao, Liang Ren, Guocheng Qi, Yukun Zhao, Zhen Xu, Long Chen, Dongmei Wang, Rui Zhang

**Affiliations:** 1 Department of Mechanics, Beijing Jiaotong University, Beijing 100044, China; 2 Beijing Composite Materials Co., Ltd., Beijing 102101, China; 3 Sinoma Science & Technology Co., Ltd., Beijing 100096, China

**Keywords:** structural power composite, bicontinuous matrices, representative volume element model, elastic–plastic behaviors

## Abstract

Due to the potential to integrate structural load bearing and energy storage within one single composite structural component, the development of carbon fiber (CF)-based structural power composites (SPCs) has garnered significant attention in electric aircraft, electric vehicles, etc. Building upon our previous investigation of the electrochemical performance of SPCs, this work focuses on elastic–plastic behaviors of the bicontinuous structural electrolyte matrices (BSEMs) and carbon fiber composite electrodes (CFCEs) in SPCs. Representative volume element (RVE) models of the BSEMs were numerically generated based on the Cahn–Hilliard equation. Furthermore, RVE models of the CFCEs were established, consisting of the BSEM and randomly distributed CFs. The moduli of BSEMs and the transverse moduli of CFCEs with different functional pore phase volume fractions were predicted and validated against experimental results. Additionally, the local plasticity of BSEMs and CFCEs in the tensile process was analyzed. The work indicates that the presence of the bicontinuous structure prolongs the plasticity evolution process, compared with the traditional polymer matrix, which could be used to explain the brittle-ductile transition observed in the matrix-dominated load-bearing process of CFCEs in the previous literature. This work is a step forward in the comprehensive interpretation of the elastic–plastic behaviors of bicontinuous matrices and multifunctional SPCs for realistic engineering applications.

## 1. Introduction

Structural power composites (SPCs) [1,2,3] based on carbon fibers (CFs), a new multifunctional composite material, have the potential to integrate structural load bearing and energy storage within one single structural composite component, which enables further mass savings in structural composites and increases the cruise endurance of electric vehicles and electric aircrafts. SPCs are promising in engineering structures with the use of traditional carbon fiber-reinforced composites and electricity energy storage devices, such as satellite panels, aircraft skins and frames, and various irregularly shaped structures [4]. SSC structural components, as a demonstration of SPC technology readiness, have been fabricated, such as automotive boot lids [5] or an aircraft door [6].

In our previous work, the electrochemical performance of SPCs was extensively investigated [3,7,8]; however, their mechanical performance is equally critical. Therefore, this study aims to elucidate their mechanical behavior. The design and fabrication of SPCs require the careful selection and integration of key components, including structural CF electrodes, structural electrolytes (SEs), and separators [9]. Two promising solutions, high internal phase emulsion templating and reaction-induced phase separation, have been proposed for fabricating SEs with specific dual-phase microstructures [10,11]. Generally, typical dual-phase structures [12] are comprised of two continuous phases that form a rather complex percolating network microstructure, featuring a bicontinuous phase of liquid electrolyte intermingled with a cross-linked polymer matrix [13]. From a multifunctional perspective, the bicontinuous structure in SEs ensures efficient ionic conduction between electrodes and mechanical load transferring in all directions [14]. Thus, bicontinuous structural electrolyte matrices (BSEMs) are employed as multifunctional matrices in SPCs. In the experimental preparation, the initial mixture of BSEM components is entirely homogeneous before resin curing. The structural epoxy solid phase and functional pore phase form during the curing process due to phase separation. The structural epoxy solid phase serves as a structural backbone for load transfer within the composite, while the functional pore phase filling with liquid facilitates ion transport [15].

Myungeun et al. [16] found that once the mass percentages of the liquid phase exceeded 30 wt%, the mechanical behaviors of SPCs transitioned from brittle to ductile. Zhou et al. [17] also found that the ionic liquid content in BSEMs significantly influenced both the microstructural phase structures and the matrix-dominated mechanical properties of the carbon fiber composite electrodes (CFCEs), including transverse tensile, short beam shear, and flexural properties. In particular, the ‘brittle to ductile’ transition was observed by increasing the ionic liquid content to a certain value. CFCEs with low ionic liquid content exhibited typical composite-like brittle behavior, while those with higher ionic liquid content showed more pronounced yielding plateaus in matrix-dominated responses such as flexure [18,19], shear [20], and transverse tensile [21,22,23].

So far, to the best of the authors’ knowledge, the theoretical structure-property relationships of the BSEMs and the corresponding SPCs have not been comprehensively interpreted. Essentially, theoretical research based on computational methods should be conducted on elastic and plastic behaviors of BSEMs and CFCEs, considering the bicontinuous matrices’ characteristics at the micromechanical level. In order to micromechanically investigate, the advanced computational modelling techniques should be introduced for generating the bicontinuous matrices. The methods for generating bicontinuous structures can be classified into three categories [24]: the geometrical method [25,26], tomographic reconstruction [27,28], and the physics-based method [29,30,31,32]. The geometrical method employs Gaussian random fields and level cut functions to construct statistically representative virtual structures. This approach provides strong controllability and is well-suitable for early-stage design; however, it does not capture the underlying physical mechanisms. Tomographic reconstruction, based on FIB-SEM technology, achieves high-fidelity reproduction of real microstructures by sequentially scanning and reconstructing three-dimensional samples. Nevertheless, it requires expensive equipment, involves complex sample preparation, and is destructive. In contrast, the physics-based method, which simulates phase separation using the Cahn–Hilliard equation, not only captures the actual microstructure evolution of polymer resin and liquid electrolyte components but also provides high versatility, making it suitable for theoretical modeling and performance prediction of multiphase materials.

Therefore, this work focuses on investigating the elastic-plastic behavior of BSEMs and CFCEs and interpreting the underlying mechanical mechanisms in SPCs. The RVE models of BSEMs and CFCEs with different geometric parameters were generated numerically based on the Cahn–Hilliard equation. The elastic-plastic response of BSEMs was subsequently simulated and analyzed, and the matrix-dominated transverse tensile stress-strain behavior of CFCEs was examined. The numerical results coincided well with the mechanical testing results of the prepared BSEMs and CFCEs. This work could provide a new insight into understanding the elastic-plastic behaviors of the multifunctional SPCs via advanced computational modelling methods.

## 2. Experimental

### 2.1. Materials

The carbon fiber used was Toray T700 (Toray Industries, Inc., Tokyo, Japan) unidirectional carbon fabrics with an areal density of 300 g/m^2^. The epoxy resins used in this study were diglycidyl ether of bisphenol A (DGEBA) epoxy resin E51 (Wuxi Bluestar Resin Co., Ltd., Wuxi, China) and tetrafunctional epoxy resin AG-80 (Shanghai Huayi Resin Co., Ltd., Shanghai, China). The D400 polyetheramine (Huntsman Corporation, Arlington, TX, USA) was used as the hardener. The liquid electrolyte ingredients were 1-ethyl-3-methylimidazolium bis (trifluoromethylsulfonyl) imide (EMIM-Tf_2_N, 99%, ShangHai Cheng Jie Chemical Co., Ltd., Shanghai, China), propylene carbonate (PC, 99%, Kuer Chemistry Co., Ltd., Hefei, China), and lithium bis (trifluoromethanesulfonyl) imide (LiTf_2_N, 99.9%, Sigma-Aldrich, St. Louis, MO, USA).

### 2.2. Specimen Preparation

The liquid electrolyte component was prepared with LiTf_2_N dissolved in the mixture of 99 wt% EMIM-Tf_2_N and 1 wt% PC. E51, AG80, and the liquid electrolyte were blended and placed in an oven at 55 °C for 5 min. The mixture was then stirred at 65 °C for 30 min using a magnetic stirrer in an oil bath to ensure uniform mixing. D400 hardener was added into the homogenous solution, followed by a mixing and degassing process. The curing cycle for BSEM was 80 °C for 2 h and 110 °C for 4 h. The mass ratio of the solid epoxy to liquid electrolyte was 1:1. Further details on the BSEMs could be referred to in Ref. [33].

The CFCEs were fabricated via a vacuum-assisted resin infusion process. Notably, the T700 carbon fiber fabrics were used without any de-sizing treatment prior to fabrication. The original surface sizing was retained to ensure an adequate fiber-matrix interfacial adhesion, which is critical for mechanical performance. Unidirectional T700 carbon fiber fabrics were stacked layer by layer in the same fiber orientation on a flat mold. A vacuum bag was then applied over the stacked fabrics, and the edges were carefully sealed. The epoxy resin–ionic liquid mixture was infused into the carbon fiber layup under vacuum. The impregnated layup was then cured in an oven under vacuum conditions, following the same curing cycle as that used for the BSEMs.

### 2.3. Characterization

The specimens for Scanning Electron Microscope (SEM) observation were submerged in ethanol, which was changed every 12 h to extract the liquid phase. After seven days, the specimens were dried in the oven (Model No: SN-DZF-6090B(Zhejiang Lichen Instrument Technology Co., Ltd., Shaoxing, Zhejiang, China)) at 75 °C under vacuum until each sample mass was constant. The morphology was observed using the CamScan JEOL 6010 (JEOL Ltd., Akishima, Japan).

BSEMs tensile tests were conducted for mechanical evaluation according to ASTM D638. The specimens were dumbbell-shaped with a total length of 115 mm, total width of 19 mm, parallel length of 33 mm, parallel width of 6 mm, transition radius of 14 mm, and thickness of 5 mm. Strain was measured using an extensometer (Model No: MTS 634.25F-24 (MTS Systems Co., Ltd., Eden Prairie, MN, USA); gauge length: 50 mm). The tensile strength and modulus were measured at a crosshead speed of 5 mm/min using Instron 5966 (Instron Corporation, Ltd., Norwood, MA, USA) with a 5 kN load cell. CFCEs tensile tests were performed according to ASTM D3039 using Instron 5982 (Instron Corporation, Norwood, MA, USA) at a crosshead speed of 5 mm/min with a 300 kN load cell. The specimens had a length of 175 mm, a width of 25 mm, and a thickness of 2 mm. Strain was measured using strain gauges (Model No: BX120-3AA (Huangyan Juxing Electrical Measurement Components Co., Ltd., Taizhou, China)). Stress-strain data were recorded during testing to evaluate the mechanical properties of both the BSEMs and CFCEs.

## 3. Modelling

### 3.1. Phase Separation Modelling Theory

The Cahn–Hilliard equation [34] was originally proposed from a phase-separation phenomenon that used the spinodal decomposition in binary alloys. Spinodal decomposition is a phase separation mechanism in which a homogeneous mixture becomes unstable and spontaneously separates into distinct phases without requiring nucleation sites. Unlike classical nucleation, which requires overcoming a thermodynamic barrier, spinodal decomposition is driven solely by diffusion and occurs uniformly throughout the material [35]. Due to its inherent simplicity, the model has widespread use in other fields [36,37], such as phase separation, multiphase fluid flow, and image inpainting.

The Cahn–Hilliard equation can be written as:

(1) $$\frac{\partial c}{\partial t}-\nabla \cdot M\left(\nabla \left(f(c)-\lambda \nabla^{2}c\right)\right)=0$$

where *c* is the concentration of one phase in the mixture, *t* is time, *M* is the mobility (usually take the constant 1), *λ* is a constant related to the thickness of the interfacial regions between phases, and *f*(*c*) represents the free energy density of a homogeneous material of concentration *c*, which can be obtained from the total free energy *F*(*c*):

(2) $$F\left(c\right)=\int_{V}\left[f\left(c\right)+\frac{1}{2}\kappa {\left(\nabla c\right)}^{2}\right]\;dv$$

where *κ* is the gradient energy coefficient, and *f*(*c*) is usually by the double-well potential:

(3) $$f\left(c\right)=\zeta c^{2}{\left(1-c\right)}^{2}$$

where *ζ* is a positive constant determining the magnitude of the energy barrier between two equilibrium phases.

### 3.2. Numerical Model of BSEMs

The numerical implementation of the finite difference algorithm for the solution of the conserved Cahn–Hilliard equation [38] was adapted into a script that could be run in the commercial finite element (FE) software Abaqus (version number: 2022). The Python script, which integrated the finite difference algorithm of the Cahn–Hilliard equation with the model generation procedure, was executed via the ***File*** → ***Run Script*** function in Abaqus. Through a series of Boolean operations within the ***Assembly*** module, the BSEMs were efficiently constructed. In the subsequent simulations, a ***Static, General*** analysis step was used, with the equation solver set to ***Direct*** and the solution technique specified as ***Full Newton***. The models were discretized using ***tetrahedral elements*** (***Tet***), and displacement-controlled loading was applied. In the post-processing stage, contour plots and numerical data were extracted from Abaqus and subsequently imported into Origin (version number: 2024b) for visualization. Stress–strain homogenization was performed by processing the reaction force-displacement data, where stress was obtained by dividing the force by the cross-sectional area, and strain was calculated by dividing the displacement by the model length.

The initial concentration directly affects the phase separation behavior. When it approaches the critical composition, the system tends to undergo spinodal decomposition, resulting in a bicontinuous and interconnected microstructure. At concentrations far from the critical point, phase separation is dominated by nucleation and growth, typically producing droplet-like morphologies. Therefore, to achieve the desired volume fraction of the functional pore phase, the initial concentrations were set to 0.4, 0.45, 0.5, 0.55, and 0.6, respectively, and the simulation was run for 2000 time steps to ensure the formation of two continuous phases. The phase separation process was modeled and terminated once the target functional pore phase volume fraction *V_fp_* was reached. Herein, *V_fp_* represents the ratio of the volume of the functional pore phase to the volume of the whole BSEM. In general, BSEMs models with five different *V_fp_* were generated, including *V_fp_* = 0.42, 0.46, 0.51, 0.55, and 0.59. Considering the stochastics, three models were generated for each *V_fp_*. The BSEM model was named in the format M0.42-1, denoting the first generated model for BSEM with *V_fp_* = 0.42.

**Figure 1:**
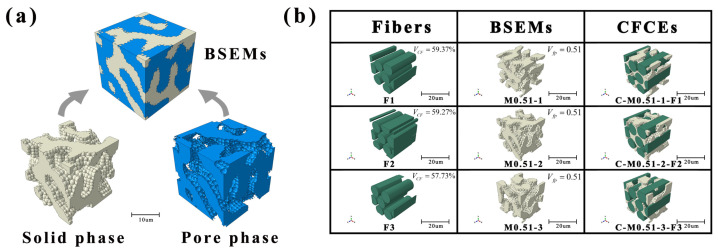
Schematic diagrams of the numerically generated models: (**a**) RVE of the BSEMs (dimensions: 20 × 20 × 20 µm^3^), (**b**) RVE of the CFCEs, including fibers (volume fraction *V_CF_*: 59.37%, 59.27%, 57.73%) and BSEMs (functional pore phase has been removed).

As shown in the M0.51-2 model Figure 1a, the RVE model has similar structures consisting of a microbead scaffold phase and a functional pore phase observed in the SEM images of BSEMs [39]. Both phases are continuous and interpenetrating. Since the functional pore phase, typically composed of liquid electrolytes or gel electrolytes, possesses negligible mechanical properties compared with the structural epoxy solid phase, only the structural epoxy solid phase was considered in the subsequent mechanical analysis. The symmetric boundary conditions were applied to three surfaces sharing the same corner point in the BSEMs RVE model. Furthermore, the mesh sensitivity analysis was conducted by comparing different mesh densities, including 0.3 μm, 0.5 μm, 1.0 μm, 2.0 μm, and 5.0 μm (which resulted in approximately 1.23 million, 1.00 million, 0.92 million, 0.80 million, and 0.50 million elements, respectively). Considering the balance between computational efficiency and accuracy, the mesh density of 1.0 μm was adopted in the FE calculations.

The material properties of the structural epoxy solid phase used were listed in Table 1. Additionally, the plastic constitutive law of BSEMs was derived from true stress and true strain data of the structural epoxy solid phase [40], and the plastic response was calibrated using the Voce model [41]. The plastic strain, *ε_p_*, can be calculated as follows:

(4) $$\varepsilon_{p}=\varepsilon -\frac{\sigma }{E_{m}}$$

where *σ* and *ε* represent the true stress and true strain obtained from the tensile experiment, respectively, and *E_m_* represents the modulus of the structural epoxy solid phase.

The Voce model was used in plasticity modelling for structural resin, shown in the following:

(5) $$\sigma =\sigma_{s}-(\sigma_{s}-\sigma_{0})\cdot e^{-C\varepsilon_{p}}$$

where *σ_0_* and *σ_s_* represent initial yield stress and saturation stress, respectively, and *C* represents the hardening rate parameter. Based on the true stress-strain data of the structural epoxy solid phase under tension, *σ_0_* was determined to be 24.72 MPa. Subsequently, Equation (5) was imported into MATLAB (Version number: 2022) for curve fitting, from which *σ_s_* and *C* were obtained as 53.40 MPa and 373.60, respectively.

### 3.3. Numerical Model of CFCEs

Based on the algorithm proposed in Ref. [43], a Python script was written to generate random CF arrays. The algorithm generated random points within a specified range to represent the centers of fiber cross-sections, with each point assigned an influence range defined by the fiber radius. Points were checked for overlap with previously generated points, and if an overlap occurred, the point was discarded and a new one was generated. This process continued until valid points were found, which were then used to create randomly distributed CFs in the FE software.

Three random CFs distributions, labeled F1, F2, and F3, were generated, as shown in Figure 1b. The carbon fiber volume fractions (*V_CF_*) in F1, F2, and F3 were 59.37%, 59.27%, and 57.73%, respectively. Subsequently, these fibers were combined with the BSEMs described in Section 2.1 via Boolean operations to generate the CFCEs model. To characterize the fiber-matrix interfacial behavior, a bilinear cohesive zone model was employed, with the interface parameters referenced from our earlier study [44,45]. The interfacial modulus, Poisson’s ratio, and thickness were 9.68 GPa, 0.374, and 0.1 μm, respectively. The failure criterion was based on the maximum interfacial stress criterion, as shown by the following equation:

(6) $$\max\left\{\frac{T_{n}^{i}}{T_{n}^{0}},\frac{T_{s}^{i}}{T_{s}^{0}}\right\}=1$$

where $T_{n}^{i}$ and $T_{s}^{i}$ represent the interfacial normal stress and axial shear stress, respectively. While $T_{n}^{0}$ and $T_{s}^{0}$ represent the normal strength and the axial shear strength, with values of 89.4 MPa and 107.5 MPa, respectively.

A total of 45 CFCE-equivalent models were constructed by combining each BSEM with three CFs. Each model was named in such a format as C-M0.42-1-F1. Where ‘C’ indicates the CFCEs, ‘F1’ indicates the CF distribution, and ‘M0.42-1’ refers to the BSEM model. The CFs distribution, BSEMs, and CFCEs RVE were shown in Figure 1b. The material properties of fibers were listed in Table 1. And the fibers were defined as linear elastic materials. The symmetric boundary conditions were the same as those used for the BSEMs. Furthermore, the mesh sensitivity analysis was conducted by comparing different mesh densities, including 0.3 μm, 0.5 μm, 1.0 μm, 2.0 μm, and 5.0 μm (mesh generation failed at densities of 0.3 μm and 0.5 μm due to limitations of the model itself; at mesh densities of 1.0 μm, 2.0 μm, and 5.0 μm, the generated models contained approximately 0.53 million, 0.45 million, and 0.20 million elements, respectively). Considering the balance between computational efficiency and accuracy, the mesh density of 2.0 μm was used in the FE calculation.

## 4. Results and Discussion

### 4.1. Experiment Results

**Figure 2:**
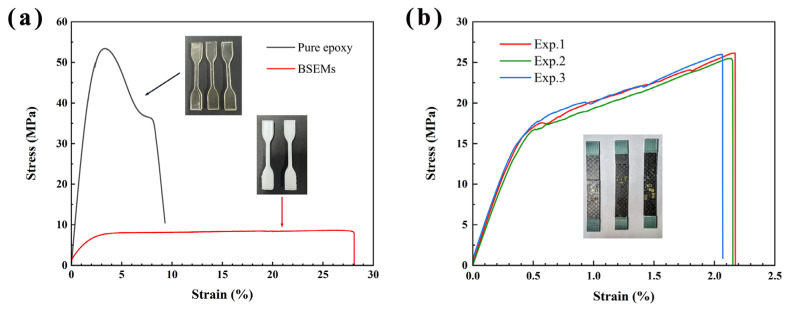
The experimental stress-strain curves: (**a**) Pure epoxy (reproduced with permission from [40], Composite Structures, 2017) and BSEMs with a functional pore phase volume fraction of 0.47 ± 0.02; (**b**) CFCEs with a functional pore phase volume fraction of 0.45 ± 0.02.

The typical measured stress-strain curves of the BSEMs and the transverse stress-strain curve of the CFCEs were plotted in Figure 2. The modulus and strength of the BSEMs (*V_fp_* = 0.47 ± 0.02, Figure 2a) were measured to be 454.48 MPa and 8.68 MPa, which are much lower than those of the pure epoxy [40] resin with a modulus of 2.83 GPa and a strength of 53.4 MPa. The modulus and strength of the CFCEs (*V_fp_* = 0.45 ± 0.02, Figure 2b) were measured to be 4.17 ± 0.11 GPa and 26.00 ± 0.01 MPa. The elongation of the BSEM was 28.1%, which is about 3.02 times that of the pure epoxy. Between the linear stage and the final breakage, a distinct ideal plastic plateau could be observed in BSEM. Compared with the tensile experimental curves of pure epoxy [40], it is evident that the BSEMs exhibit a more pronounced plastic plateau than neat epoxy resin. On the other hand, owing to the BSEM, the CFCE also exhibits a high elongation of 2.2%. This behavior is attributed to the combined effects of the relatively high-volume fraction of the functional pore phase in the bicontinuous matrix.

**Figure 3:**
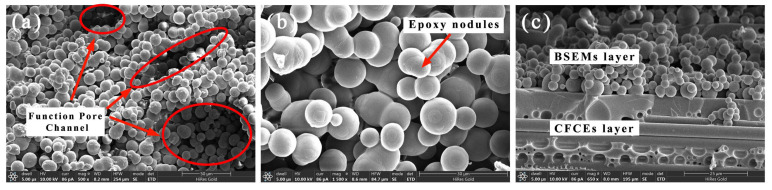
SEM images of the microstructures of: (**a**,**b**) BSEMs; (**c**) CFCEs.

As shown in the SEM images of the microstructures of BSEMs and CFCEs (Figure 3), an epoxy nodules network and functional pore channels can be clearly observed in the BSEMs (Figure 3a,b), which contribute to enhanced ultimate load-bearing capacity and improved ionic conductivity, respectively. In the CFCE (Figure 3c), the skin layer showed that the bicontinuous matrices were consistent with the bulk BSEM (Figure 3a,b), while from the surface morphology, it could be clearly seen that the CF were surrounded by the dual-phase matrix.

### 4.2. Analyses of the Mechanical Response

#### 4.2.1. Mechanical Response of BSEMs

**Figure 4:**
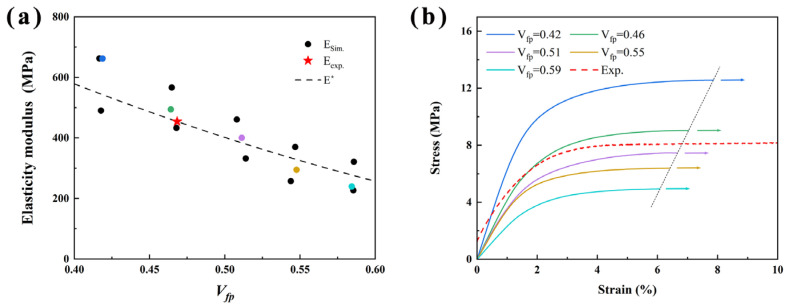
The numerical simulation mechanical properties of BSEMs with different *V_fp_* (experimental result was also plotted). The colored points in the modulus plot correspond to the colors in the stress–strain curves, representing the same model results: (**a**) Elasticity moduli and analytical solution E* [31]; (**b**) Tensile stress-strain curve (based on the fracture strain of the pure epoxy, only the results within the elastic-plastic regime were presented, as truncated by the termination curve (dotted line in the figure)).

The moduli of the BSEMs were calculated based on the homogenization method. The average modulus, ranging from 262.52 MPa to 604.96 MPa, was plotted against the functional pore volume fraction *V_fp_* in Figure 4a. As the *V_fp_* increased from 0.42 to 0.59, the average modulus of BSEMs decreased by 56.6%, indicating that the volume fraction of the functional pore phase significantly affects the modulus of BSEMs. This trend is consistent with the experimental characterization result [33] and the modelling results [31]. At high volume fractions of the functional pore phase, the pronounced reduction in modulus was attributed not only to the diminished structural epoxy solid phase but also to the development of a discontinuous matrix morphology in the BSEMs. This structural characteristic impaired load transfer efficiency and resulted in pronounced scatter in the mechanical behavior [46]. However, high *V_fp_* could impart energy storage capabilities to BSEMs. Thus, from the perspective of the multifunctional mechanism, the balance between ionic conductivity and mechanical properties should be achieved for the BSEMs [47].

In addition, the elastic moduli obtained from numerical simulation were evenly distributed around the analytical solution E* [31]. The average modulus obtained from the simulation at *V_fp_* = 0.46 was 498.0 MPa. By comparing with the experimental results (*V_fp_* = 0.47 ± 0.02, *E* = 454.48 MPa), the prediction error of the numerical model in this work was around 9.6%. Collectively, these findings validate the reliability of the BSEMs model in elastic analysis.

The numerical simulation stress-strain curves for BSEMs with different volume fractions of functional pore phase, together with the experimental curves (Exp.), were plotted in Figure 4b. It should be noted that resin fracture was not considered here, as the focus was on elastic-plastic behaviors and the limitations of computational resources. The calculations ended when the maximum principal strain exceeded the elongation of the pure epoxy.

From the curves in Figure 4b, it can be observed that as the volume fraction of the functional pore phase increases, the slope of the elastic region (corresponding to the modulus shown in Figure 4b) decreases. With strain exceeding approximately 2.0%, the stress rises gradually to a distinct plateau, indicating that most BSEMs have entered the plastic deformation stage. Carolan [31] also obtained similar plastic behaviors for the bicontinuous polymeric materials through numerical simulation. In bicontinuous structures, localized plasticity initiates early at multiple sites within the solid phase and gradually propagates throughout the entire network [31]. The increase in the functional pore phase weakens the continuity of the structural epoxy solid phase and disrupts the load-transfer paths, thereby diminishing the overall connectivity. This effect promotes the spread of plastic deformation and suppresses crack propagation, which enhances energy dissipation and leads to numerous non-catastrophic dissipative events [48].

The simulated stress-strain curves were close to the experimental result at a similar *V_fp_*. At the *V_fp_* of 0.46, the simulated yield stress is 9.04 MPa, 11.2% higher than the experiment. These results indicate that within the permissible range of experimental error, the simulation models were in good agreement with the experimental data. Overall, these numerical results suggested that the generated bicontinuous matrix models are effective and feasible for analyzing BSEMs mechanical behaviors considering influences of the volume fraction of the functional pore phase, thereby providing the fundamental basis for analyzing the mechanical behaviors of the CFCEs.

#### 4.2.2. Mechanical Response of CFCEs

**Figure 5:**
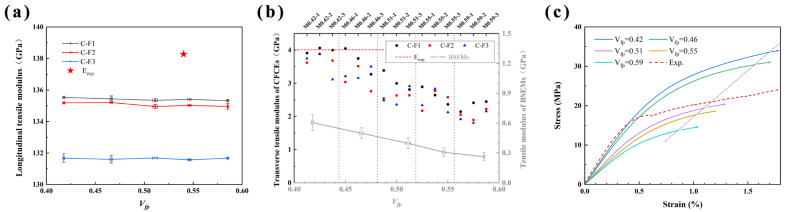
The numerical simulation mechanical properties of CFCEs with different *V_fp_* (experimental result was also plotted): (**a**) Longitudinal tensile moduli; (**b**) Transverse tensile moduli (and the tensile moduli of BSEMs were also plotted); (**c**) Transverse tensile stress-strain curve (based on the fracture strain of the pure epoxy, only the results within the elastic-plastic regime were presented, as truncated by the termination curve (dotted line in the figure)).

Figure 5a shows the longitudinal tensile moduli of CFCEs with different *V_fp_*, composed of three differently distributed carbon fibers (labeled C-F1, C-F2, and C-F3). As the *V_fp_* increases, the longitudinal tensile modulus remains relatively stable, primarily because the longitudinal tensile modulus of composites is predominantly determined by the fiber volume fraction [49]. The simulated average longitudinal tensile modulus of CFCEs was 133.6 GPa. Based on Jones’ research [50] and the data in Table 1, the theoretical longitudinal tensile modulus of CFCEs is approximately 135.6 GPa. The simulated longitudinal modulus is close to both the theoretical calculation and experimental results (*V_fp_* = 0.54 ± 0.02, *E_L_* = 138.3 GPa), being only about 1.5% lower than the theoretical value and about 3.4% lower than the experimental result. Mainly due to the incomplete reflection of matrix connectivity in the simulation. In the RVE of CFCEs, the unconnected resin part existed after combining with CF. The continuity of the structural epoxy solid phase is disrupted. Solid segments that are isolated from the surrounding fiber and matrix contribute minimally to the load-transfer process, resulting in a reduced effective modulus.

As for the transverse behavior of the carbon fiber reinforced bicontinuous matrix composites, the microstructures of the matrices play a critical role in determining the transverse moduli. Figure 5b shows the transverse tensile moduli of CFCEs from finite element simulation and experimental results, along with a line chart displaying the mean and standard deviation of the tensile moduli of BSEMs.

The transverse tensile moduli of C-F1 surpass those of C-F2 and C-F3, primarily due to differences in fiber volume fraction. The transverse tensile moduli of CFCEs are higher than those of BSEMs and decrease with increasing *V_fp_*. This effect is primarily attributed to the increase in *V_fp_*, which not only reduces the volume fraction of the structural epoxy solid phase but also exposes more surface area of the porous epoxy resin. The increased surface area weakens the interfacial bonding between fibers and the epoxy matrix, thereby impeding efficient load transfer. In the RVE of CFCEs, unconnected resin regions remain after combining with carbon fibers, disrupting the continuity of the structural epoxy solid phase. These isolated solid segments, detached from both the fibers and surrounding matrix, contribute minimally to load transfer, ultimately leading to a reduced effective modulus. When *V_fp_* = 0.4~0.5, the experimental results are consistent with those of C-F1 and slightly higher than those of C-F2 and C-F3. This validates the reliability of the CFCEs model containing F1 in modulus simulations. Therefore, the CFCEs model with F1 was used in the subsequent analyses. Similar to BSEMs, when *V_fp_* increases from 0.42 to 0.59, the average transverse tensile modulus of CFCEs containing F1 fibers decreases from 3.99 GPa to 2.34 GPa, a reduction of approximately 41.4%. This comparison reveals that the presence of fibers mitigates the impact of the reduction in pore phase content on the mechanical properties of CFCEs.

Figure 5c shows the transverse tensile stress-strain curves of CFCEs with different volume fractions of the functional pore phase, along with the experimental result (Exp.). Similar to the BSEMs, the calculation was terminated once the structural resin element satisfied the maximum strain criteria.

With the increase in *V_fp_*, the stress of CFCEs decreases at the same strain. When the transverse tensile strain is 0.8%, the stress range of CFCEs is about 13 MPa to 25 MPa, which is about twice as large as that of BSEMs. The numerical simulation indicates that BSEMs in CFCEs enter the plastic stage at a lower strain than the bulk BSEMs. Prior to a strain of 0.4%, the experimental results are in good agreement with the curves for *V_fp_* = 0.42 and *V_fp_* = 0.46. However, when the strain exceeds 0.4%, the experimental curve exhibits a distinct yield point, showing more pronounced plasticity than the numerical simulation. This discrepancy may be attributed to factors such as the presence of a resin-rich layer (the BSEMs layer in Figure 3c) in the CFCEs, providing valuable insights for future studies on the numerical simulation of structural power composites.

### 4.3. Plasticity Analysis

**Figure 6:**
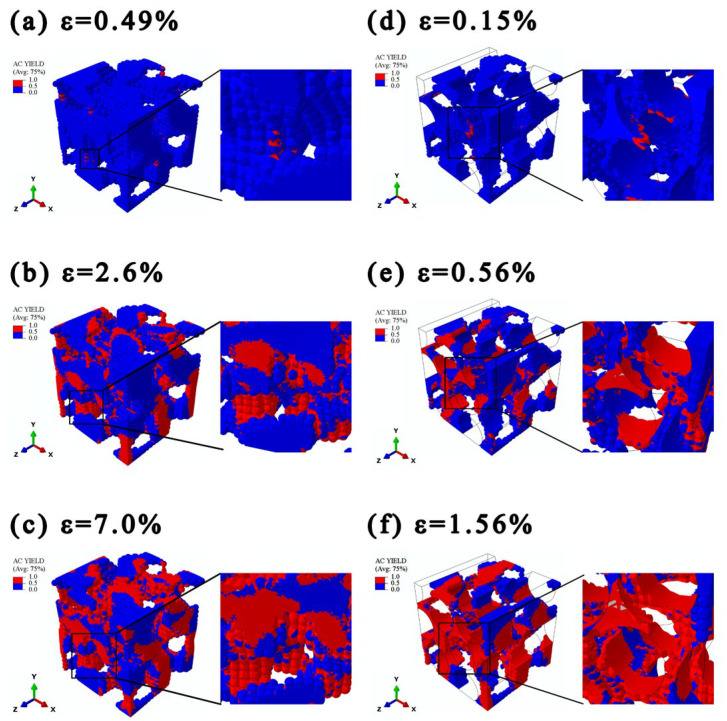
Yield state contours of BSEMs and CFCEs under different strain (plastic initiation stage, plastic propagation stage, plastic-dominated stage; the red elements represent the plastic regions, while the blue elements denote the elastic regions): (**a**–**c**) Tension of the BSEMs (M0.51-2, *V_fp_* = 0.51), (**d**–**f**) Transverse tension of the CFCEs (C-M0.51-2-F1, *V_fp_* = 0.51, *V_CF_* = 59.37%; to highlight the plastic distribution in the structural epoxy solid phase, the plastic contours of the fibers were omitted and only their outlines were retained).

**Figure 7:**
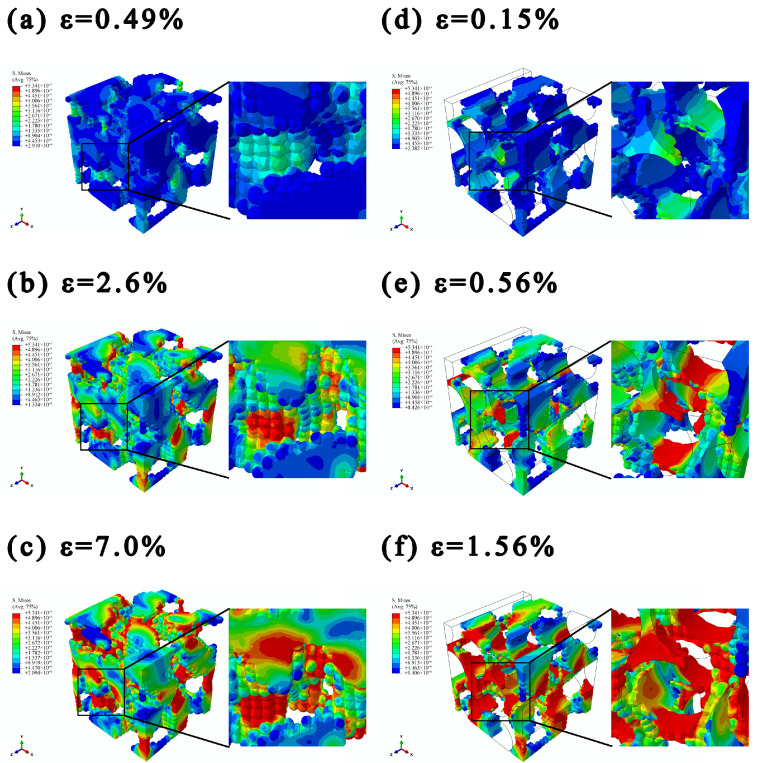
Stress contours of BSEMs and CFCEs under different strain (plastic initiation stage, plastic propagation stage, plastic-dominated stage (Unit: 10^12^ Pa): (**a**–**c**) Tension of the BSEMs (M0.51-2, *V_fp_* = 0.51), (**d**–**f**) Transverse tension of the CFCEs (C-M0.51-2-F1, *V_fp_* = 0.51, *V_CF_* = 59.37%; to highlight the stress distribution in the structural epoxy solid phase, the stress contours of the fibers were omitted and only their outlines were retained).

The yield state contours reflecting the initiation and evolution process of yielding in the BSEMs and CFCEs were shown in Figure 6. In those contours, the red elements represent plastic regions, while the blue elements denote elastic regions. And the Mises stress contours of BSEMs and CFCEs were shown in Figure 7. Based on the stress-strain curve (Figure 4b and Figure 5c), three distinct strains were selected (BSEMs: *ε* = 0.49%, *ε* = 2.6%, *ε* = 7.0%; CFCEs: *ε* = 0.15%, *ε* = 0.56%, *ε* = 1.56%), corresponding to the plastic initiation stage, the plastic propagation stage, and the plastic-dominated stage, respectively. The strain corresponding to the plastic initiation stage was determined by the elastic limit, the plastic propagation stage was defined by the yield point, and the plastic-dominated stage was identified based on the termination of the curve.

#### 4.3.1. Plasticity Analysis of BSEMs

In the plastic initiation stage (Figure 6a), i.e., at low strain levels (*ε* ≤ 0.49%), the BSEMs remain largely in an elastic state, with plasticity initiating only in localized regions. These plastic regions are primarily distributed along the boundaries of the functional pore phase or at geometric discontinuities, which are stress concentration-sensitive areas. At this stage, the stress contour (Figure 7a) exhibits a relatively uniform distribution without significant stress concentrations, indicating that the material maintains a strong overall load-bearing capacity and that its macroscopic mechanical response remains linear.

In the plasticity propagation stage (0.49% ≤ ε ≤ 2.6%, Figure 6b), the plastic region rapidly expands. Plastic elements are mainly located around the functional pore phase and gradually extend into the surrounding regions. Meanwhile, the Mises stress contour (Figure 7b) reveals several localized regions reaching high stress levels, forming multiple stress concentration cores. This stage corresponds to the nonlinear transition region in the stress-strain curve shown in Figure 4b, indicating a reduction in the overall stiffness of the BSEMs and marking the onset of the yielding transition.

In the plasticity-dominated stage (ε ≥ 7.0%, Figure 6c), with further strain increase, the plastic region propagates throughout nearly the entire structural epoxy solid-phase skeleton. The coverage of plastic elements expands significantly, indicating that the BSEMs have entered a state of large-scale plastic flow.

As shown in Figure 7a–c, with increasing *V_fp_*, the matrix connectivity decreased and the local cross-sectional area was reduced, leading to a significant increase in the local Mises stress. Consequently, stress concentration was primarily localized at the narrow neck regions along the loading direction. At a strain of 0.49%, the structure was dominated by low-stress areas with only a few isolated high-stress spots. When the strain reached 2.6%, the fractions of low-stress and medium-stress regions became comparable, indicating a progressively heterogeneous stress distribution. At 7.0% strain, high-stress areas had expanded, leaving only a few low-stress areas, while the proportion of medium-stress regions slightly exceeded that of the high-stress ones. Notably, the maximum von Mises stress reached 53.4 MPa, which is consistent with the tensile strength of the structural epoxy solid phase, confirming that the simulation results remained within the elastic-plastic regime.

As the applied load increased, the narrow neck regions initially experienced their maximum stresses. With further loading, stress levels in the remaining low-stress regions began to rise, whereas the previously localized high-stress areas remained nearly constant rather than continuing to increase. This redistribution and progressive propagation of stress across the entire structural epoxy solid phase occurred without immediate structural failure. Such a gradual and spatially diffused stress accumulation mechanism effectively delayed overall failure, thereby enabling the BSEMs to exhibit substantially enhanced plasticity compared with pure epoxy.

#### 4.3.2. Plasticity Analysis of CFCEs

The calculation stops when the maximum principal strain exceeds the elongation limit of pure epoxy. Since the interfacial normal strength $T_{n}^{0}$ and axial shear strength $T_{s}^{0}$ are 89.4 MPa and 107.5 MPa, respectively, both higher than the tensile strength of CFCEs (26.0 MPa), the simulation is terminated before significant interfacial debonding or failure occurs. Therefore, the interfacial effects on the mechanical performance of CFCEs in the system we simulated are negligible.

The yield state contours of CFCEs show that during the plastic initiation stage (*ε* = 0.15%, Figure 6d), plastic deformation in the CFCE initiation occurs at the interface between the carbon fibers and the structural epoxy solid phase. As the strain increases to 0.56% (Figure 6e), the plastic region rapidly propagates along the fiber direction. As the strain further increases to 1.56% (Figure 6f), the plastic region expands across most of the structural epoxy solid phase, exhibiting a distinct pattern of regional propagation. This evolution indicates that CFCEs undergo plastic deformation at relatively low strain levels, with the plastic region predominantly distributed along the fiber alignment.

A comparison with the plastic evolution process of BSEMs reveals that CFCEs enter the plastic stage earlier than BSEMs and exhibit a faster expansion of the plastic region. This difference primarily arises from the incorporation of carbon fibers, which disrupts the continuity of the structural epoxy solid phase, leading to the formation of numerous discontinuous matrix regions. These isolated solid-phase areas lack stable stress transfer pathways during loading, leading to stress concentration and consequently causing earlier plastic deformation in the CFCEs.

Further comparison between the Mises stress contours of BSEMs (Figure 7a–c) and CFCEs (Figure 7d–f), the incorporation of fibers markedly altered the stress concentration distribution within the structural epoxy solid phase: the localized stress concentrations shifted from the narrow neck regions to a distribution predominantly along the fiber direction. This shift indicates that the presence of fibers facilitates load redistribution within the structural epoxy solid phase and modifies the loci of stress concentration. Compared to BSEMs, CFCEs reached the plastic limit earlier and more rapidly, indicating that the presence of fibers enhances load transfer efficiency and accelerates the progression of plastic deformation. In conventional composites, the circumferential stress distribution along the fiber-matrix interface exhibits a well-defined periodic pattern, with stress maxima typically occurring at azimuthal angles parallel and antiparallel to the loading direction, while minima are observed at the perpendicular orientations. In contrast, the CFCEs display a noticeable angular shift in the locations of these stress extrema.

As loading continues, the plastic region progressively expands into surrounding elastic regions, driven by high stress gradients, which illustrates a characteristic mechanism of ‘progressive plasticity accumulation’. Therefore, this underlying mechanism explains the distinct plateau observed in the stress-strain curve. Such a deformation mechanism also provides a micromechanical explanation for the observed brittle-to-ductile transition in CFRP [17] as the ionic liquid content increases, where the microstructural changes promote more distributed plasticity and delay catastrophic failure.

## 5. Conclusions

This work is aimed at investigating the elastic-plastic response in the load-bearing process of BSEMs and CFCEs. The numerical model of BSEMs and CFCEs was created based on the Cahn–Hilliard equation. An increase in *V_fp_* resulted in a reduction of the moduli of BSEMs and the transverse tensile moduli of CFCEs. When *V_fp_* increases from 0.42 to 0.59, the average modulus of BSEMs decreases from 662.7 MPa to 226.8 MPa, corresponding to a reduction of approximately 56.6%, whereas the average transverse tensile modulus of CFCEs containing F1 fibers decreases from 3.99 GPa to 2.34 GPa, a reduction of about 41.4%. This suggests that the presence of fibers mitigates the influence of *V_fp_* on the modulus of CFCEs. Compared to pure resin matrix, BSEMs exhibited a more pronounced plasticity. The maximum tensile strain of BSEMs reaches 28.1%, which is approximately three times higher than that of pure epoxy at 9.20%. BSEMs could undergo substantial shape changes, reflecting their high ductility and energy absorption capacity as the structural load bearing. Additionally, stress and plasticity contours indicate that localized plastic deformation in BSEMs initiates early at multiple sites and gradually propagates throughout the structure. In contrast, the plastic region of CFCEs is primarily concentrated near the fibers. In the BSEMs, plastic deformation initiates at a strain of ε = 0.49% and extends to nearly the entire structural epoxy solid phase at ε = 7.0%. In contrast, for the CFCEs, plastic deformation initiates earlier at ε = 0.15% and extends to almost the entire structural epoxy solid phase by ε = 1.56%. These results demonstrate that, compared with BSEMs, CFCEs exhibit an earlier onset of plasticity and a significantly faster propagation rate. The progressive plasticity of the bicontinuous structure leads to the brittle-ductile transition of BSEMs and matrix-dominated performances of CFCEs. The work offers a comprehensive analysis of the influences of *V_fp_* on the elastic moduli and plasticity behaviors of CFCEs based on the 3D computational micromechanics models of emerging SPCs. Valuable insights are provided into the load transfer mechanisms between the fiber and the matrix, which are crucial for mechanically optimizing SPCs elastic-plastic behaviors from tailoring the functional pore phase of BSEMs. In addition to clarifying the structure-multifunctional relationships of dual-phase systems, the micromechanical model considering the phase-separated microstructure of BSEMs also opens up opportunities for optimizing the pore phase content in both BSEMs and CFCEs to achieve balanced mechanical and electrochemical performance.

## Figures and Tables

**Table 1:** Mechanical properties of the T700 carbon fiber and the structural epoxy solid phase. (Reproduced with permission from [40], Composite Structures, 2017, and [42], Composites Science and Technology, 2007).

T700	Parameters	Structural Epoxy Solid Phase	Parameters
Longitudinal tensile modulus $E_{fL}(\mathrm{GPa})$	230.00	Tensile modulus $E_{m}\left(\mathrm{GPa}\right)$	2.83
Transverse tensile modulus $E_{fT}(\mathrm{GPa})$	23.10	Poisson’s ratio $\upsilon_{m}$	0.35
Longitudinal shear modulus $G_{fLT}\left(\mathrm{GPa}\right)$	8.96	Tensile strength $X_{mT}\left(\mathrm{MPa}\right)$	53.40
Transverse shear modulus $G_{fTT}(\mathrm{GPa})$	8.27	Density $\rho_{m}({g/\mathrm{cm}}^{3})$	1.20
Longitudinal (main) Poisson’s ratio $\upsilon_{fLT}$	0.20	Maximum tensile strain	9.20%
Transverse (Secondary) Poisson’s ratio $\upsilon_{fTT}$	0.40		
Density $\rho_{f}{(g/\mathrm{cm}}^{3})$	1.80		
Fiber radius $r_{f}\left(\mu m\right)$	3.50		

## Data Availability

Data is contained within the article.

## Abbreviations

The following abbreviations are used in this manuscript:

SPCs	structural power composites
CFs	carbon fibers
SEs	structural electrolytes
BSEMs	bicontinuous structural electrolyte matrices
CFCEs	carbon fiber composite electrodes
SEM	Scanning Electron Microscope
FE	finite element
